# Quantitative Analysis of Glutathione and Carnosine Adducts with 4-Hydroxy-2-nonenal in Muscle in a hSOD1^G93A^ ALS Rat Model

**DOI:** 10.1021/acs.chemrestox.4c00052

**Published:** 2024-07-27

**Authors:** Pablo V. M. Reis, Bianca S. Vargas, Rafael A. Rebelo, Mariana P. Massafera, Fernanda M. Prado, Hector Oreliana, Henrique V. de Oliveira, Florêncio P. Freitas, Graziella E. Ronsein, Sayuri Miyamoto, Paolo Di Mascio, Marisa H. G. Medeiros

**Affiliations:** Departamento de Bioquímica, Instituto de Química, Universidade de São Paulo, São Paulo, SP 05508-900, Brazil

## Abstract

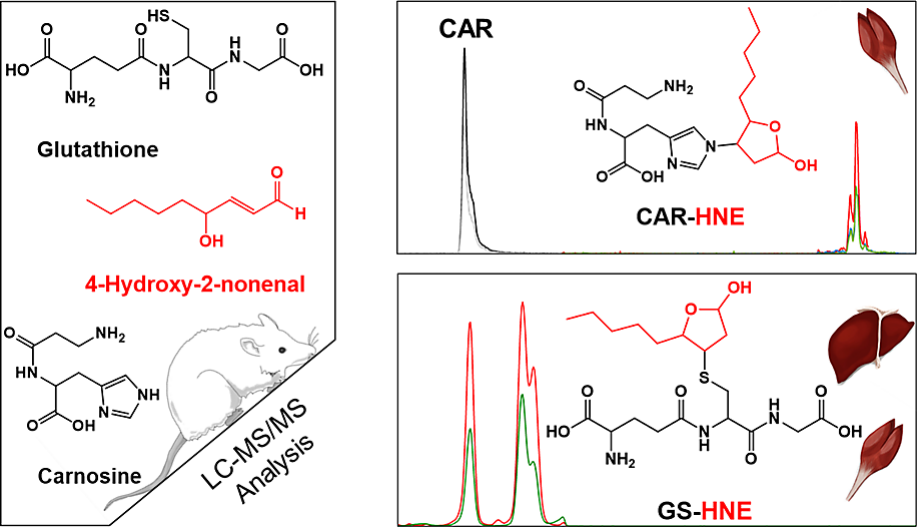

Amyotrophic lateral sclerosis (ALS) is a fatal neurodegenerative disease characterized by the dysfunction and death of motor neurons through multifactorial mechanisms that remain unclear. ALS has been recognized as a multisystemic disease, and the potential role of skeletal muscle in disease progression has been investigated. Reactive aldehydes formed as secondary lipid peroxidation products in the redox processes react with biomolecules, such as DNA, proteins, and amino acids, resulting in cytotoxic effects. 4-Hydroxy-2-nonenal (HNE) levels are elevated in the spinal cord motor neurons of ALS patients, and HNE-modified proteins have been identified in the spinal cord tissue of an ALS transgenic mice model, suggesting that reactive aldehydes can contribute to motor neuron degeneration in ALS. One biological pathway of aldehyde detoxification involves conjugation with glutathione (GSH) or carnosine (Car). Here, the detection and quantification of Car, GSH, GSSG (glutathione disulfide), and the corresponding adducts with HNE, Car-HNE, and GS-HNE, were performed in muscle and liver tissues of a hSOD1^G93A^ ALS rat model by reverse-phase high-performance liquid chromatography coupled to electrospray ion trap tandem mass spectrometry in the selected reaction monitoring mode. A significant increase in the levels of GS-HNE and Car-HNE was observed in the muscle tissue of the end-stage ALS animals. Therefore, analyzing variations in the levels of these adducts in ALS animal tissue is crucial from a toxicological perspective and can contribute to the development of new therapeutic strategies.

## Introduction

Amyotrophic lateral sclerosis (ALS) is a fatal neurodegenerative disease characterized by progressive loss of motor neurons, leading to muscle atrophy, paralysis, and, ultimately, death.^1^ While the majority of cases are sporadic with unknown causes, approximately 10% are familial inherited forms (FALS).^2^ Of these FALS cases, around 25% are associated with mutations in the gene encoding cytosolic Cu, Zn-superoxide dismutase (SOD1), a highly abundant antioxidant enzyme in the central nervous system.^3^ Notably, there is no clear correlation between enzyme activity, clinical progression, and disease phenotype, as most mutants retain full catalytic activity.^4,5^ One extensively studied mutation is the Gly^93^ → Ala (G93A), primarily due to the availability of SOD1^G93A^ transgenic animals.^6^ These animals, including mice and rats overexpressing the human Gly^93^ → Ala (G93A) mutant enzyme, display features similar to ALS patients, such as progressive motor neuron degeneration.^7^ It is known that SOD1 mutants linked to FALS tend to form toxic aggregates leading to oxidative molecular damage and mitochondrial dysfunction, ultimately triggering apoptosis in neuronal cells.^8^ In addition to neuronal effects, ALS patients also suffer from systemic inflammation, resulting from pro-inflammatory signaling that permeates the blood-brain barrier.^9^ Serum levels of acute phase proteins have been related to disease progression, indicating liver involvement in the disease burden.^10^ Oxidative stress biomarkers like 3-nitrotyrosine, 8-hydroxy-2′-deoxyguanosine and *trans*-4-hydroxy-2-nonenal (HNE) have been found to be elevated in ALS patients.^11^ Additionally, HNE-protein adducts have been observed to be elevated in brain tissue and body fluids in various age-related neurodegenerative diseases, including Alzheimer’s, Parkinson’s, and Huntington’s diseases and ALS subjects, or in corresponding disease models.^12^ Endogenously, reactive aldehydes, including HNE, 2,4-decadienal, malondialdehyde, 4-oxo-2-nonenal, 4,5-epoxy-2-decenal, hexenal, 2-propenal (acrolein), and crotonaldehyde, are formed as secondary lipid peroxidation products.^13^ Many of these aldehydes react with biomolecules, such as DNA, proteins, and amino acids, resulting in cytotoxic effects and contributing to various disease and aging processes.^14−16^ The types of aldehydes formed during lipid peroxidation depend on the polyunsaturated fatty acids present in the membrane, as observed by Kawai et al.^17^ HNE is particularly well-studied due to its high reactivity with a wide range of biomolecules.^18^ It exerts cytotoxic effects, such as inhibiting enzyme activity and protein, DNA, and RNA synthesis. HNE also induces the expression of heat shock proteins and plays a role in inhibiting cell proliferation.^19^ Furthermore, HNE has been shown to possess genotoxic and mutagenic properties.^19−21^ It is widely recognized that HNE is a strong electrophile, preferentially reacting with compounds containing thiol groups (e.g., cysteine, glutathione, SH-containing proteins) and exhibiting lower reactivity with compounds containing amino groups.^22^ Aldehydes are enzymatically detoxified by alcohol dehydrogenase, aldo–keto reductase, and aldehyde dehydrogenase.^23^ Reactive aldehyde detoxification in living cells also involves conjugation with glutathione (GSH) to form Michael adducts. GSH, found at millimolar concentrations in mammalian cells, is a defense against redox stress. The combination of GSH with aldehydes, catalyzed by glutathione-S-transferase (GST) is a widely studied process. GSTs are present in both eukaryotic and prokaryotic organisms and catalyze reactions with various substrates forming GS-adducts.^23^

Additionally, endogenous histidine-containing dipeptides, such as carnosine (β-alanyl-l-histidine, CAR), homocarnosine (*gamma*-amino-butyryl-histidine), and anserine (β-alanyl-L-1-methylhistidine), have also been recognized as detoxifying agents against reactive carbonyl species.^24^ Carnosine is present in high concentrations in skeletal muscle and central nervous system.^24^

**Scheme 1:**
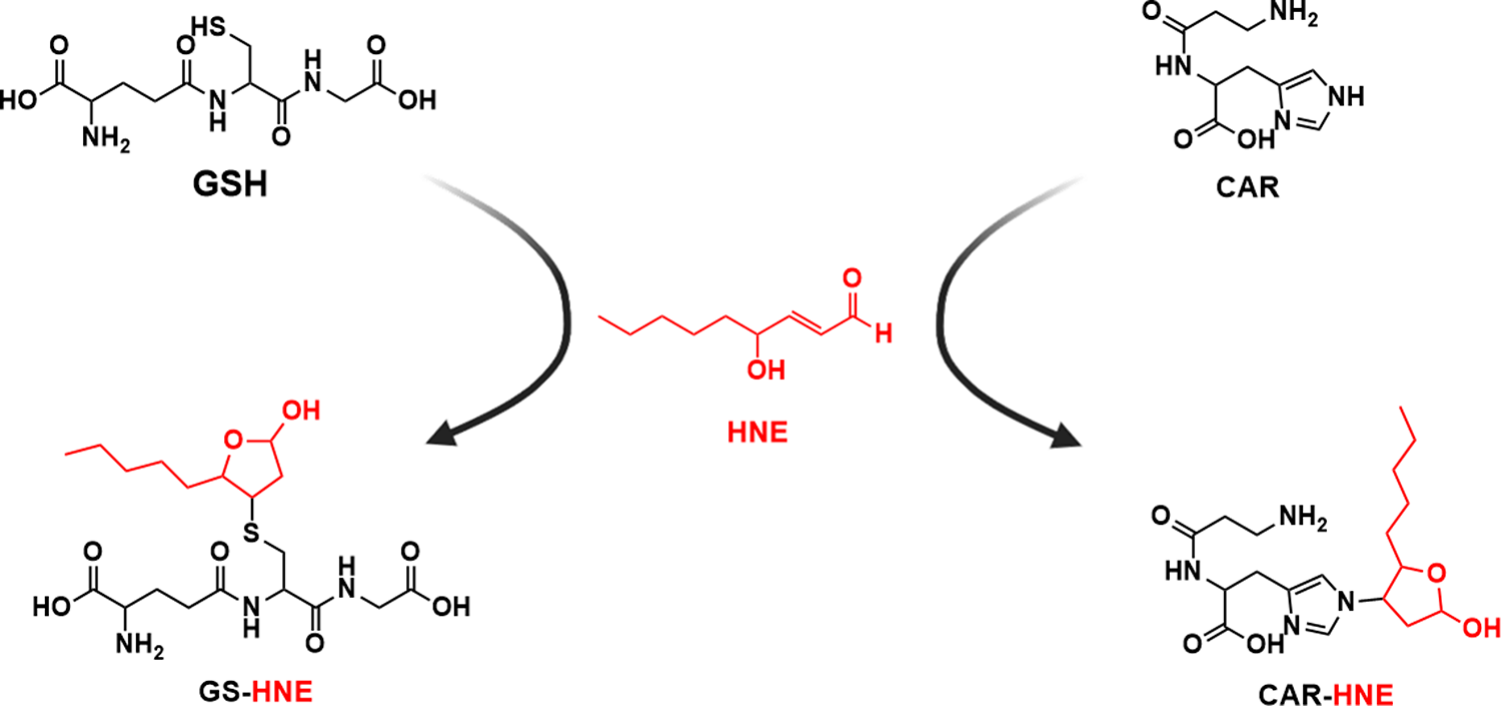
Detoxification Products of *trans*-4-Hydroxynonenal Reaction with Either Glutathione or Carnosine

Recently, ALS has been recognized as a multisystemic disease, involving structural and metabolic changes in various cell types that contribute to the progression of the disease. A new potential role of skeletal muscle in this scenario has been investigated.^25,26^ In this study, we utilized liver and skeletal muscle (gastrocnemius) to gain a better understanding of the redox distress that occurs during disease progression. The biological quantification of Car, GSH, GSSG, and the adducts of both peptides with HNE (Scheme 1) in muscle samples from an hSOD1^G93A^ ALS rat model was conducted using online reverse-phase HPLC coupled with electrospray mass spectrometry.

## Experimental Procedures

### Materials and Methods

#### Chemicals

All chemicals were of the highest commercially available purity grade. Water was purified on a Milli-Q system (Millipore, Bedford, MA). GSH, GSSG, Car, and HNE and further reagents were acquired from Sigma-Aldrich (St. Louis, MO, USA) or Merck (Darmstadt, Germany). [^13^C_2_,^15^N-Gly]-glutathione, l-Carnosine-*d*_4_, and 4-hydroxynonenal-*d*_11_ were acquired from C/D/N Isotopes Inc. (Quebec, CA). All solvents were HPLC grade and were purchased from Sigma-Aldrich. Solutions were prepared in a Milli-Q water.

#### Animals

The animal study was conducted in accordance with the ethical principles for animal experimentation, according to the guidelines of the National Council for Animal Experimentation Control (Conselho Nacional de Controle de Experimentação Animal—CONCEA, Ministry of Science, Technology, Innovation and Communications, Brazil). All animal procedures were approved by the University of Sao Paulo Chemistry Institute’s Animal Care and Use Committee under protocol number 194/2021. Male Sprague–Dawley rats overexpressing human SOD1^G93A^ were obtained from Taconic and bred with wild-type Sprague–Dawley females to establish a colony.^27^ Genotyping by PCR to detect exogenous hSOD1 transgenes was performed using DNA extracted from ear punching at 20 days of age. Rats were housed under controlled laboratory conditions, including room temperature, a 12/12 h light/dark cycle with food and water ad libitum. Asymptomatic ALS rats (*n* = 12) and their wild-type controls (*n* = 12) were sacrificed at 70 days of age, while symptomatic ALS rats (*n* = 16), end-stage ALS rats (*n* = 8), and their controls (*n* = 12) were sacrificed at 120 days of age. The criterion for symptomatic ALS was the appearance of hind-limb weakness and for the end-stage was the hind-limb paralysis and 15% weight loss.^28^ Before euthanasia, rats were fasted for 6 h and anesthetized with 4% isoflurane.

#### GSH and GSSG Extraction from Rat Tissues

The tissues (50 mg liver and 30 mg muscle) were homogenized in 2 mL of buffer A (320 mM sucrose, 5 mM MgCl_2_, 10 mM Tris–HCl, 0.1 mM deferoxamine, 1% Triton X-100, pH 7.5) with 15.2 pmol of internal standard ([^15^N, ^13^C_2_-Glycines]—GSSG). The solution was centrifuged at 1300 × *g* and 9 °C for 10 min. 100 μL were collected for protein quantification using a Pierce BCA Protein Assay Kit, Thermo Scientific. Another 300 μL of supernatant were collected and 240 μL of derivatization solution [20 mM N-ethylmaleimide (NEM), 2 mM EDTA, and 1 mM deferoxamine] were added. The solution was stirred for 1 min and kept on ice for 30 min. After that, the proteins were precipitated by the addition of 60 μL of cold 5% TCA, stirred for 1 min, kept on ice for 10 min, and centrifuged at 12,000 × *g*, 9 °C for 10 min. The remaining fat in the supernatant was extracted by adding 200 μL of CHCl_3_ followed by stirring for 1 min and centrifugation at 12,000 × *g* for 10 min at 9 °C, and this process was repeated 3 times. Finally, the upper phase was subjected to filtration in 0.1 μm poly(vinylidene difluoride) (PVDF) Centrifugal Filters (Millipore, Bedford, MA, USA) through centrifugation at 12,000 × *g*, 9 °C for 10 min. For the analysis in the HPLC-MS/MS system, 20 μL of the samples were injected for the quantification of GSSG. For GSH quantification, the sample was further diluted 200 times and the same volume was used for analysis.

#### GS-HNE Extraction from Rat Tissues

The muscle or liver tissue was removed, washed with ice-cold physiological saline solution, and frozen at −80 °C. Subsequently, the tissue was cooled with liquid nitrogen and subjected to lyophilization for better preservation and homogenization. The sample (200 mg liver, 90 mg muscle) was homogenized in 2 mL TKM buffer (50 mM Tris–HCl pH 7.5, 25 mM KCl, 5 mM MgCl_2_). 5.76 pmol amount of internal standard ([^13^C_2_,^15^N-Gly]-GS-HNE), 2 mL of 1 mM PBS pH 7.0, and 150 μL of 2% butyl hydroxytoluene (BHT) were added. The solution was stirred for 1 min and kept on ice for 10 min. 100 μL were collected for further protein quantification using a Pierce BCA Protein Assay Kit, Thermo Scientific. Next, proteins were precipitated by the addition of 1 mL cold 5% TCA, stirred for 1 min, kept on ice for 10 min, and centrifuged at 11,000 × *g*, 9 °C for another 10 min. The remaining fat in the supernatant was extracted three times by addition of 1 mL of CHCl_3_, stirred for 1 min, and centrifuged at 11,000 × *g*, 9 °C for 10 min. The resulting solution (4 mL) was filtered in 0.1 μm PVDF Centrifugal Filters (Millipore, Bedford, MA, USA), concentrated using a Refrigerated CentriVap Concentrator (Labconco, Kansas City, USA), resuspended in 115 μL of water, and 20 μL of the solution were analyzed by mass spectrometry.

#### Carnosine and Car-HNE Extraction from Rat Muscle Samples

The analytes were extracted as previously reported^29^ first using a PowerGen 1000 homogenizer (Thermo Fisher Scientific, Waltham, MA) to process 50 mg of lyophilized gastrocnemius sample with 1.5 mL of the extraction buffer consisting of (150 mM KH_2_PO_4_, 1 mM EDTA, 1 mM DTT and diluted (1:1000) protease inhibitor cocktail (Sigma Chemical Co., St. Louis, MO)). After homogenization, samples were centrifuged at 12,000 × *g* for 20 min at 9 °C, and the pellet was discarded. The supernatant (30 μL) was aliquoted for protein quantification using the Pierce BCA Protein Assay Kit (Thermo Fisher Scientific, Waltham, MA), according to the manufacturer’s instructions.

Protein precipitation was carried out by adding 3% HClO_4_ (70% v/v) to the supernatant, stirring for 5 s, and then a 15 min ice bath rest. Samples were then centrifuged for 10 min at 12,000 × *g* and 9 °C, resulting once again in a pellet to be discarded and a working supernatant. The pH of the supernatant was increased to 5.5 using 10 M NaOH and the solution was filtered through Ultrafree—MC Durapore PVDF 0.1 μm Centrifugal Filters (Merck Millipore Ltd., Cork, Ireland), by a 10 min centrifugation at 12,000 × *g* and 9 °C. 500 μL of each sample were dried using a Refrigerated CentriVap Concentrator (Labconco, Kansas City, USA) at 4 °C and resuspended in 100 μL solution of 1.3 × 10^–8^ M Car-HNE*d*_11_. 80 μL were injected into the HPLC system. For carnosine quantification, 10 μL of the remaining volume was diluted 10,000×. To 50 μL of the resulting solution, 5 μL of 1 × 10^–6^ M Car*d*_4_ was added and 20 μL was used for each analysis.

#### Synthesis and Purification of the GS-HNE Internal Unlabeled and the Isotopically Labeled Internal Standards ([^13^C_2_,^15^N-Gly]-GS-HNE)

Both adducts were synthesized and purified according to the protocols described by Falletti et al.^30^ The GS-HNE adducts were purified using a Shimadzu LC-6AD HPLC system (Shimadzu, Kyoto, Japan), which included a Rheodyne injector (Cotati, CA) and a SPD-M10AVP photodiode array detector, controlled by a SCL-10A/V communication module and CLASS VP software. HPLC separations were conducted in a Luna C18(2) (250 × 10 mm, 5 μm particle size, 100 Å) semipreparative column (Phenomenex, Torrance, CA) with a gradient of 0.1% formic acid in water (A) and acetonitrile (B) used as follows: 10–30% B (0–40 min), 30–50% B (40–45 min), 50% B (45–50 min), 50–10% B (50–55 min), and 10% B (55–60 min) to re-equilibrate the column. The flow rate was 5 mL/min. Absorbance was monitored at 220 nm. The 26–30 min eluate was collected, lyophilized, and kept at −20 °C. Both adducts were characterized by ^1^H NMR and reverse-phase high-performance liquid chromatography coupled electrospray tandem mass spectrometry in positive mode (HPLC-ESI^+^-MS/MS).^28^ HPLC-ESI^+^-MS/MS analyses, carried out into a triple quadrupole Quattro II mass spectrometer (Micromass, Manchester, UK), generated mass spectra containing protonated and dehydrated ions of GS-HNE ([M + H]^+^*m*/*z* = 464, M + H – H_2_O]^+^*m*/*z* = 446) and [^13^C_2_,^15^N-Gly]-GS-HNE ([M + H]^+^*m*/*z* = 467, [M + H – H_2_O]^+^*m*/*z* = 449), which were consistent with literature data.^31^

#### Synthesis, Purification, and Analysis of the CAR-HNE and CAR-HNE*d*_11_ Internal Unlabeled and the Isotopically Labeled Standards

CAR-HNE and CAR-HNE*d*_11_ were prepared, purified, and analyzed as described before.^32^

#### Quantification of GSH (GS-NEM) and GSSG in Rat Tissues

Online HPLC-ESI^+^-MS/MS analyses were carried out in the positive mode, and detection was conducted on a triple quadrupole API 4000 QTRAP mass spectrometer (Applied Biosystems, Foster City, CA), using selected reaction monitoring (SRM). The turbo ionspray voltage was kept at 5000 V, curtain gas at 10 psi and nebulizer and auxiliary gas at 50 psi. The temperature was set at 500 °C, and pressure of nitrogen in the collision cell was adjusted to high. An Agilent HPLC system (Agilent Technologies, Santa Clara, CA) equipped with an autosampler (1200 High Performance), a column oven set at 20 °C (1200 G1216B), an automated high-pressure flow switching valve, a 1200 Binary Pump SL and an Isocratic Pump (1200 SL G1310A) was used for sample injection using a Luna C18 column (150 mm × 2.0 mm i.d., 3 μm particle size; Phenomenex, Torrance, CA, USA) maintained at 20 °C. Mobile phases were 0.1% (v/v) formic acid in water (A), 0.1% (v/v) formic acid in acetonitrile (B) and a mixture of A:B 1:1 (v/v) (C), flow rate at 200 μL/min. Prior to use, solutions were filtered through a 0.1 μm PVDF membrane (Millipore, Bed-ford, MA). GS-NEM and GSSG were eluted from the column according to the following method: from 0 to 5 min, 1% B; from 5 to 15 min, 1 to 40% B; from 15 to 20 min, 40 to 95% B; from 20 to 24 min; 95% B; from 24 to 25 min, 95 to 1%; from 25 to 35 min, 1% to re-equilibrate the column, flow rate at 200 μL/min. A high-pressure flow switching valve composed of 2-positions and 6-ports was inserted after the column. The valve discarded the eluate from the column until 5 min of run while kept the mass spectrometer supplied with solvent C at a constant flow of 50 μL/min using the isocratic pump. After 5 min of run, the valve switched position allowing the eluate from the column to enter the mass spectrometer. After 13 min of run, the valve switched back to waste position.

#### Quantification of GS-HNE in Rat Tissues

Online HPLC-ESI^+^-MS/MS analyses were carried out in the positive mode, and detection was conducted on a triple quadrupole mass spectrometer API 6500 (Sciex, Framingham, MA), using SRM. The turbo ionspray voltage was kept at 5500 V, curtain gas at 20 psi, and nebulizer and auxiliary gas at 50 psi. The temperature was set at 550 °C, and nitrogen pressure in the collision cell was adjusted to high. An Agilent HPLC system (Agilent Technologies, Santa Clara, CA) equipped with an autosampler (1200 High Performance), a column oven set at 30 °C (1200 G1216B) with automated high-pressure flow switching valve, a 1200 Binary Pump SL (1200 G1310A) and a Shimadzu 10-AVp Isocratic Pump (Shimadzu, Tokyo, Japan) was used for sample injection using a Luna C18 column (150 mm × 2.0 mm i.d., 3 μm particle size; Phenomenex, Torrance, CA, USA) maintained at 20 °C, the flow rate was 250 μL/min. Mobile phases were 0.1% (v/v) formic acid in water (A), formic acid 0.1% (v/v) in acetonitrile (B), and a mixture of A:B 1:1 (v/v) (C). Prior to use, solutions were filtered through a 0.1 μm PVDF membrane (Millipore, Bedford, MA). The adducts were eluted from the column according to the following method: from 0 to 5.5 min, 10% B; from 5.5 to 10 min, 10 to 15% B; from 10 to 21 min, 15% B; from 21 to 23 min; 15 to 50% B; from 23 to 26 min, 50%; from 26 to 33 min, 50 to 90% B; from 33 to 36 min; 90% B; from 36 to 46 min; 90 to 10% B; from 46 to 55 min, 10% B to re-equilibrate the column. A high-pressure flow switching valve composed of 2-positions and 6-ports was inserted after the column. The valve discarded the eluate from the column until 5.5 min of run while keeping the mass spectrometer supplied with solvent C at a constant flow of 100 μL/min using a Shimadzu 10-AVp Isocratic Pump. After 5.5 min of run, the valve switched position allowing the eluate from the column to enter the mass spectrometer. After 13 min of run, the valve switched back to the waste position. After 17 min the valve position is switched again, allowing the eluate from the column to enter the mass spectrometer. After 26 min of run, the valve switched back to the waste position. MS^2^ spectrum was performed using the same conditions described in this section and in Table S1 (Supporting Information).

#### Quantification of Carnosine and CAR-HNE in Rat Muscle

HPLC-ESI^+^-MS/MS analyses were conducted on a triple quadrupole QTRAP 6500 mass spectrometer (Sciex, Framingham, MA), using SRM. The adducts were analyzed by electrospray ionization (ESI) in positive mode, and detection was made using SRM. The turbo ion spray voltage was kept at 5500 V, curtain gas at 20 psi, and nebulizer and auxiliary gases at 40 psi. The temperature was set at 500 °C, and the pressure of nitrogen in the collision cell was adjusted to high. The HPLC oven was set to 35 °C, keeping two columns: Kinetex C18 100 × 4.6 mm inner diameter, particle diameter of 2.6 μm (Phenomenex, Torrance, CA) followed by Kinetex C18 100 × 2.1 mm inner diameter, particle diameter of 2.6 μm (Phenomenex, Torrance, CA) and the valve between them at the same temperature. The mobile phase consisted of three solutions, 5 mM ammonium acetate pH 5.5 (A), acetonitrile (B) and 99.5% 5 mM ammonium acetate pH 5.5, and 0.5% acetonitrile (C). Prior to use, aqueous phases were filtered through a 0.1 μm PVDF membrane (Millipore, Bedford, MA).

The analytes were eluted from the columns according to the following method: from 0 to 10 min, a 99.5% A flow increased from 200 to 300 μL/min; then decreased to 250 μL/min in the next 36 s. From 10.6 to 20 min, 99.5 to 0.5% A, maintaining both flow and phase concentration for another 5 min. From 25 to 28 min, 250 to 200 μL/min, and 99.5% A, conditions were maintained until the end of the run, at 40 min. The valve was kept open only between 3 and 23 min, feeding the second column and the mass spectrometer with the C phase for the remaining parts of the analysis. MS^2^ spectrum was performed using the same conditions described in this section and in Table S2 (Supporting Information).

#### Statistical Analysis

Statistical analyses were performed using GraphPad Prism version 5 for Windows (GraphPad Software, San Diego California USA). Comparisons between the two groups were performed with Student’s *t* test. For multiple groups, non-normal distributed data were transformed and an ANOVA test with Tukey’s Multiple Comparison post-test was performed. Results were considered significant for values of *p < 0.05; **p < 0.01. NS = not significant.

#### Mass Spectrometry Analysis

Signal-to-noise ratio S/*N* ≥ 7 was used as the quantification criteria for all analytes. Analyst version 1.6.2 was used for acquisition and peak integration. Tables S1 and S2 show the *m*/*z* and mass spectrometry parameters for each analyte.

## Results and Discussion

### GS-HNE Quantification in Rat Tissues

**Figure 1:**
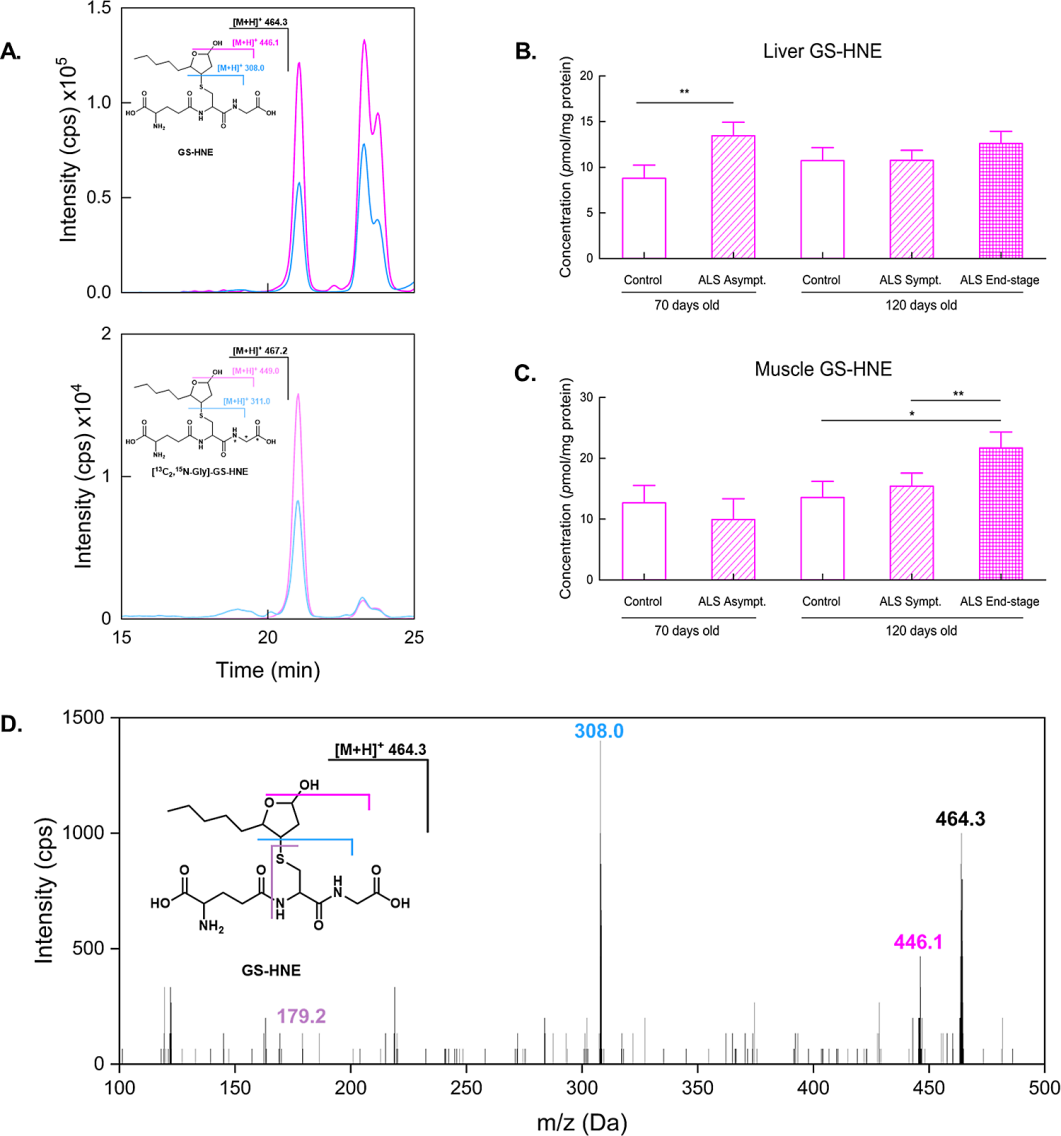
Representative chromatograms of the GS-HNE adducts in the liver of a control rat (A). The endogenous adduct (top) exhibits two equally intense peaks, representing biologically formed isomers. The labeled internal standard (lower) predominantly forms the first peak, indicating a difference in isomeric formation between in vitro and in vivo systems. GS-HNE levels in liver (B) and muscle tissue (C) of 70 and 120-day-old ALS rats along with the corresponding controls. Full MS^2^ of the GS-HNE adduct, showing the two fragments monitored in SRM mode and *m*/*z* 179.2 (D).

HNE is produced as a secondary byproduct of the lipid peroxidation process. Reactive aldehydes have the capability to modify biomolecules including DNA, proteins, and peptides.^19−21^ As previously mentioned, HNE levels have been shown to be increased in patients with ALS.^11^ A well-established pathway of aldehyde detoxification involves their conjugation with GSH. The intracellular GSH concentration typically falls within the range of 3–4 mM^20^ and, as discussed by Blair,^31^ the difference in concentrations between aldehydes and GSH in cells facilitates GSH detoxification mechanisms. Therefore, levels of the GS-HNE adduct can be useful to monitor redox stress in tissues in the course of the disease. To achieve this, we employed a sensitive methodology based on HPLC-ESI^+^-MS/MS for the precise quantification of GS-HNE adduct in the muscle of both control and ALS rats. The methodology enables direct analyte quantification, which is formed through the reaction of GSH with HNE (Scheme 1). Figure 1 shows the chromatographic peak profiles for endogenous and internal standard GS-HNE (A) and GS-HNE quantification in liver (B) and muscle (C) of ALS rats and controls. Only representative chromatograms for liver analysis are shown due to an equal peak profile in both analyzed tissues. Additional confirmation of adduct identity is shown (Figure 1D) by full MS^2^ spectrum analysis. These data are in accordance with Orioli et al.^33^

Levels of the GS-HNE adduct showed a significant increase in the liver of ALS asymptomatic 70-day-old rats when compared to their respective controls. As the disease progressed, the levels of GS-HNE in the livers of 120-day-old rats did not display any significant difference compared to controls of the same age, neither in the symptomatic nor in the end-stage animals. Given that GSH is particularly concentrated in the liver and HNE is one of the main aldehydes generated during the lipid peroxidation process, the observed elevation in GS-HNE adduct levels in asymptomatic 70-day-old rats may indicate an increase in the redox stress during this stage of the disease.

When assessed in rat *gastrocnemius* muscles, a significant increase in GS-HNE was observed in ALS end-stage animals compared with ALS symptomatic and 120-day-old control individuals. This significant increase in the GS-HNE adduct in rat gastrocnemius muscles indicates an important rise in the levels of HNE, reflected by an elevation in its detoxification mechanism by GSH.

**Figure 2:**
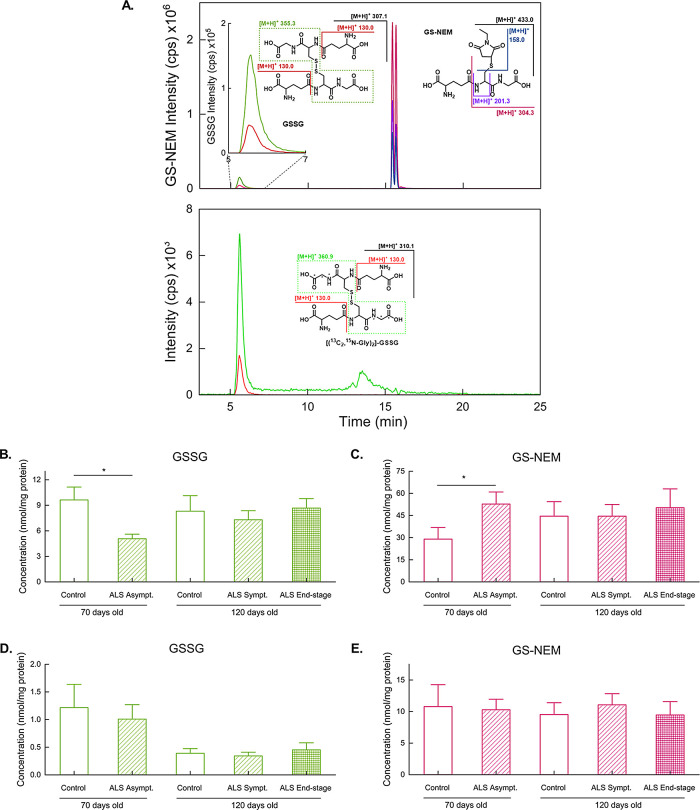
Representative chromatograms of GSSG and the GS-NEM adduct in the liver of a control rat (A). The reduced form of glutathione forms two equally intense peaks, indicating biological isomers. Endogenous (upper) and internal standard (lower) peaks for GSSG show the same profile, indicating that isomers formed both in vivo and in vitro. GSSG (B) and GS-NEM (C) levels in the liver. GSSG (D) and GS-NEM (E) levels in the muscle tissue of 70 and 120-day-old ALS rats along with the corresponding controls.

To ensure that the differences in GS-HNE presence were not due to GSH availability, the same isotopical dilution technique was performed to assess GSH/GSSG equilibrium using [^15^N, ^13^C_2_-Glycines]–GSSG as the internal standard, as shown in Figure 2.

The GSH and GSSG levels in end-stage animals showed no significant change in either liver or muscle in any of the analyzed groups. Only representative chromatograms for liver analysis are shown due to an equal peak profile in both tissues. For comparative purposes, the GSH/GSSG ratio is shown in Figure S1 (Supporting Information). This behavior is expected, given that the concentration of GSH in tissues is much higher than that of HNE. An observed significant increase in liver GSH levels of 70-day-old rats suggests a redox stress response in this stage of the disease.

Lipid-derived electrophilic compounds such as HNE exhibit high reactivity toward nucleophilic groups in proteins and other biomolecules. Simpson et al.^34^ described that serum and cerebrospinal fluid HNE levels were significantly elevated in sporadic (sALS) cases compared to healthy control groups. Further, in patients with sALS, serum HNE increased over time and correlated positively with advancing disease.

### CAR-HNE and Carnosine Adduct in Muscle Tissue

**Figure 3:**
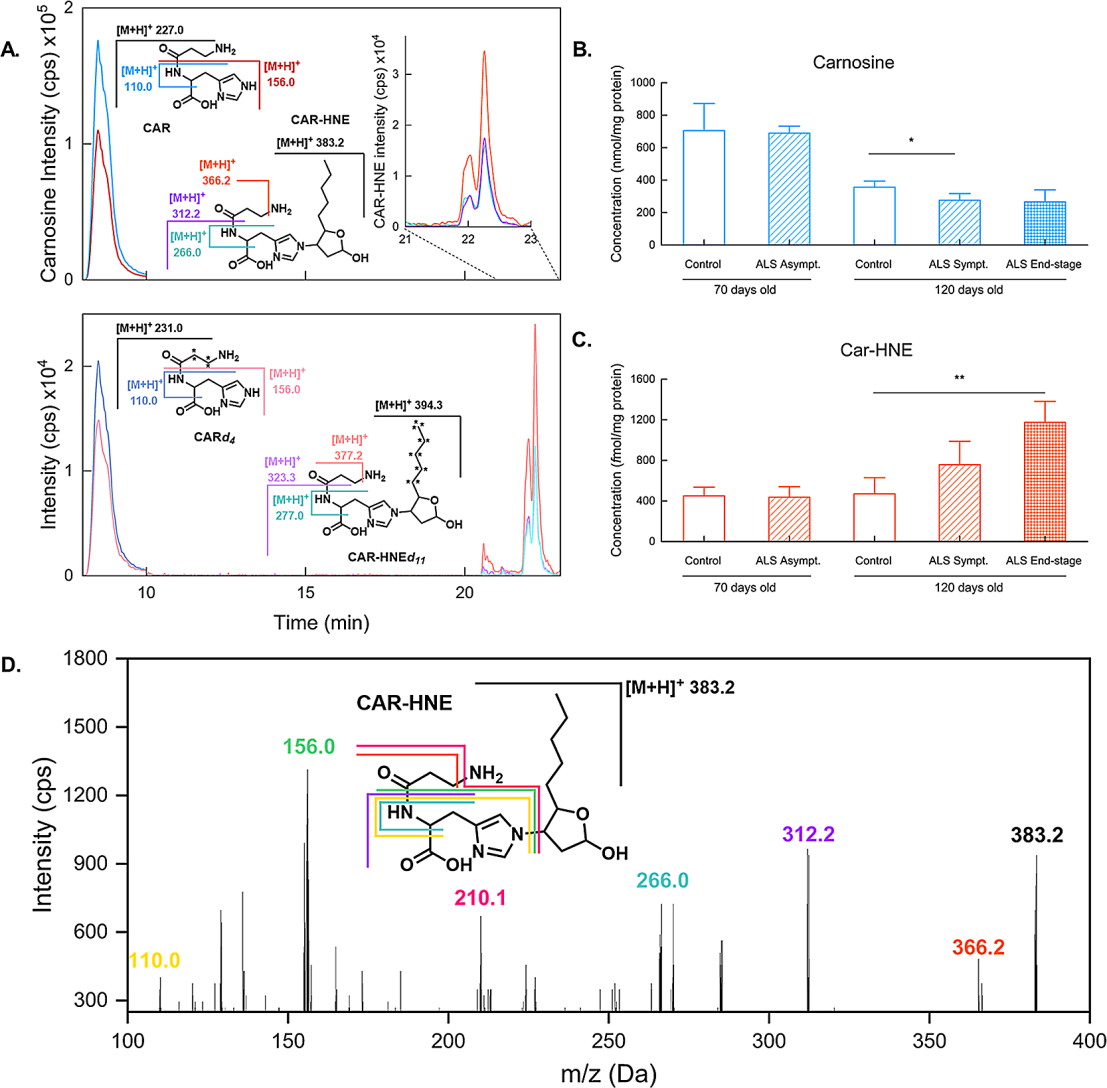
Representative chromatogram for control rat muscle sample, showing successful Carnosine and CAR-HNE separation, in both endogenous analytes (upper) and internal standards (lower) (A). Car-HNE shows two peaks in both in vivo and in vitro synthesis, indicating that endogenous (upper) and internal standard (lower) forms are composed of the same isomers. Levels of Carnosine (B) and Car-HNE (C) in muscle tissue of 70 and 120-day-old ALS rats along with corresponding controls. Full MS^2^ of the Car-HNE adduct (D).

Figure 3 shows a representative chromatogram for CAR-HNE and carnosine detection in *gastrocnemius* samples (A). Additional confirmation of adduct identity was obtained by full MS^2^ spectrum, as shown in Figure 3D, in accordance with Orioli et al.^33^

Interestingly, carnosine levels in the muscle of control rats decreased with age (Figure 3B). This suggests that carnosine levels are more susceptible to decrease in the face of muscle loss. A statistically significant decrease was also observed in the symptomatic 120-day-old ALS rat muscle compared to controls at the same age. This is in accordance with data described by Stuerenburg and Kunze,^35^ who found decreased carnosine tissue concentrations in muscle biopsies of ALS patients and in rats. In our experimental conditions, in accordance with Boldyrev,^36^ Car was not detected in liver samples.

In line with the findings for muscular GS-HNE, increased levels of Car-HNE were also found in end-stage animals (Figure 3C). This observation suggests an increase in HNE formation in the group and underscores the effect of detoxification mechanisms exerted by GSH and carnosine.

It is notable that despite carnosine (500 nmol/mg of protein) being present at a concentration about 50 times higher than that of GSH (10 nmol/mg of protein), the levels of GS-HNE are about 20 times greater than those of the CAR-HNE adduct, indicating that GSH is a more efficient scavenger of HNE in this tissue. This is in accordance with the bimolecular rate constant values for the formation of the two adducts (0.035 M^–1^ s^–1^ for the reaction of HNE with carnosine and 1.09 M^–1^ s^–1^ for the reaction with GSH).^37,38^ Furthermore, the contribution of glutathione transferase (GST) to the formation of GS-HNE cannot be dismissed.

## Conclusions

Here, using a very sensitive LC–MS/MS methodology, levels of GSH and Car adducts with HNE were quantified in the liver and muscle of ALS SOD1^G93A^ transgenic rats. The increase in HNE detoxification products following aging and disease progression suggests augmented lipid peroxidation in the muscle tissue.

Recently, ALS has been considered a multisystem disorder with changes in structural and metabolic parameters in different cell types that synergistically contribute to the disease progression.^26^ Recent studies are investigating whether skeletal muscle plays a critical role in the pathology of ALS.^39^ Mastrogiovanni and colleagues^40^ found a significant decrease in oxylipins derived from linoleic acid in the plasma of ALS patients, including 13-hydroperoxy-9,11-octadecadienoic acid (13-HODE). It has also been described that plasma levels of 13-HODE and 9-hydroxy-octadecadienoic acid (9-HODE) positively correlate with the duration of the disease. The breakdown of 13-HODE generates HNE, inducing carbonyl stress.^41^

The results described here point to an increase in the detoxification products of HNE in the late phase of the disease, suggesting an increase in redox stress in the muscles of rats with ALS. Considering the cytotoxic role of reactive aldehydes, the increased levels of the GS-HNE adduct in the muscle deserve more attention for a better understanding of pathological changes and how this stress impacts the disease. Understanding these mechanisms may contribute to the development of new therapeutic strategies.

## Supporting Information Available

Mass spectrometry parameters for detection and quantification of glutathione analytes; quantification transitions are highlighted; mass spectrometry parameters for detection and quantification of carnosine analytes; quantification transitions are highlighted; mass spectrometry parameters for detection and quantification of free 4-hydroxynonenal analytes; quantification transitions are highlighted; GS-NEM and GSSG ratio in the analyzed tissues; free 4-hydroxynonenal extraction from rat muscle tissue; quantification of HNE-DNPH in rat tissues; analysis of HNE-DNPH and HNE*d*_11_-DNPH (internal standard) derivatives in muscle tissue; HPLC/ESI^–^/MS–MS analyses carried out in the negative mode (PDF)

## Abbreviations

ALS: amyotrophic lateral sclerosis
Car: carnosine
GSH: glutathione
GSSG: glutathione disulfide
HNE: 4-hydroxy-2-nonenal
GS-HNE: adduct between GSH and HNE
Car-HNE: adduct formed between Car and HNE
HPLC/MS–MS: high-performance liquid chromatography/tandem mass spectrometry

## References

[ref1] Hardiman O.; Al-chalabi A.; Chio A.; Corr E. M.; Logroscino G.; Robberecht W.; Shaw P. J.; Simmons Z.; van den Berg L. H. Amyotrophic Lateral Sclerosis. Nat. Rev. Dis. Primers 2017, 3, 71 10.1038/nrdp.2017.71.

[ref2] Ingre C.; Roos P. M.; Piehl F.; Kamel F.; Fang F. Risk Factors for Amyotrophic Lateral Sclerosis. Clin. Epidemiol. 2015, 7, 181–193. 10.2147/CLEP.S37505. 25709501 PMC4334292

[ref3] Rosen D. R.; Siddique T.; Patterson D.; Figlewicz D. A.; Sapp P. II; Hentati A.; Donaldson D.; Goto J.; O’Regan J. P.; Deng H.; Rahmani Z.; Krizus A.; Mckenna-yasek D.; Cayabyab A.; Gaston S. M.; Berger R.; Tanzi R. E.; Halperin J. J.; Herzfeldt B.; Van den Bergh R.; Hung W.; Bird T.; Deng G.; Mulder D. W.; Smyth C.; Laing N. G.; Soriano E.; Pericak-Vance M. A.; Haines J.; Rouleau G. A.; Gusella J. S.; Horvitz H. R.; Brown Jr R. H. Mutations in Cu/Zn Superoxide Dismutase Gene Are Associated with Familial Amyptrophic Lateral Sclerosis. Nature 1993, 362, 59–62. 10.1038/362059a0. 8446170

[ref4] Borchelt D. R.; Lee M. K.; Slunt H. S.; Guarnieri M.; Xu Z.; Wong P. C.; Brown R. H.; Price D. L.; Sisodia S. S.; Cleveland D. O. N. W. Superoxide Dismutase 1 with Mutations Linked to Familial Amyotrophic Lateral Sclerosis Possesses Significant Activity. Proc. Natl. Acad. Sci. U. S. A. 1994, 91, 8292–8296. 10.1073/pnas.91.17.8292. 8058797 PMC44592

[ref5] Rabizadeh S.; Gralla E. B.; Borchelt D. R.; Gwinn R.; Valentine J. S.; Sisodia S.; Wong P.; Lee M.; Hahn H.; Bredesen D. E. Mutations Associated with Amyotrophic Lateral Sclerosis Convert Superoxide Dismutase from an Antiapoptotic Gene to a Proapoptotic Gene: Studies in Yeast and Neural Cells. Proc. Natl. Acad. Sci. U. S. A. 1995, 92, 3024–3028. 10.1073/pnas.92.7.3024. 7708768 PMC42351

[ref6] Gurney M. E.; Pu H.; Chiu A. Y.; Canto M. C. D.; Polchow C. Y.; Alexander D. D.; Caliendo J.; Hentati A.; Kwon Y. W.; Deng H.; Chen W.; Zhai P.; Sufit R. L.; Siddique T. Motor Neuron Degeneration in Mice That Express a Human Cu, Zn Superoxide Dismutase Mutation. Science 1994, 264, 1772–1775. 10.1126/science.8209258. 8209258

[ref7] Bendotti C.; Carrı M. T. Lessons from Models of SOD1-Linked Familial ALS. Trends Mol. Med. 2004, 10 (8), 393 10.1016/j.molmed.2004.06.009. 15310460

[ref8] Cozzolino M.; Ferri A.; Carri M. T. Amyotrophic Lateral Sclerosis: From Current Developments in the Laboratory to Clinical Implications.. Antioxid. Redox Signaling 2008, 10 (3), 1918 10.1089/ars.2007.1760. 18370853

[ref9] Appel S. H.; Beers D. R.; Zhao W. Amyotrophic Lateral Sclerosis Is a Systemic Disease: Peripheral Contributions to Inflammation- Mediated Neurodegeneration. Curr. Opin. Neurol. 2021, 34, 765–772. 10.1097/WCO.0000000000000983. 34402459

[ref10] Beers D. R.; Zhao W.; Neal D. W.; Thonhoff J. R.; Thome A. D.; Faridar A.; Wen S.; Wang J.; Appel S. H. Elevated Acute Phase Proteins Reflect Peripheral Inflammation and Disease Severity in Patients with Amyotrophic Lateral Sclerosis. Sci. Rep. 2020, 10, 1–17. 10.1038/s41598-020-72247-5. 32943739 PMC7499429

[ref11] Barber S. C.; Mead R. J.; Shaw P. J. Oxidative Stress in ALS: A Mechanism of Neurodegeneration and a Therapeutic Target. Biochim. Biophys. Acta 2006, 1762, 1051–1067. 10.1016/j.bbadis.2006.03.008. 16713195

[ref12] Di Domenico F.; Tramutola A.; Butterfield D. A. Role of 4-Hydroxy-2-Nonenal (HNE) in the Pathogenesis of Alzheimer Disease and Other Selected Age-Related Neurodegenerative Disorders. Free Radicals Biol. Med. 2017, 111, 253–261. 10.1016/j.freeradbiomed.2016.10.490. 27789292

[ref13] Medeiros M. H. G. DNA Adducts as Biomarkers of Lipid Oxidation and Predictors of Disease. Challenges in Developing Sensitive and Specific Methods for Clinical Studies. Chem. Res. Toxicol. 2009, 55, 419–425. 10.1021/tx800367d. 19166334

[ref14] Ullery J. C.; Marnett L. J. Protein Modification by Oxidized Phospholipids and Hydrolytically Released Lipid Electrophiles: Investigating Cellular Responses. Biochim. Biophys. Acta - Biomembr. 2012, 1818 (10), 2424–2435. 10.1016/j.bbamem.2012.04.014. PMC3398744 22562025

[ref15] Medeiros M. H. G. DNA Damage by Endogenous and Exogenous Aldehydes. J. Braz. Chem. Soc. 2021, 30 (10), 2000–2009. 10.21577/0103-5053.20190056.

[ref16] Barrera G.; Pizzimenti S.; Daga M.; Dianzani C.; Arcaro A.; Cetrangolo G. P.; Giordano G.; Cucci M. A.; Graf M.; Gentile F. Lipid Peroxidation-Derived Aldehydes, 4-Hydroxynonenal and Malondialdehyde in Aging-Related Disorders. Antioxidants 2018, 7 (8), 102 10.3390/antiox7080102. 30061536 PMC6115986

[ref17] Kawai Y.; Takeda S.; Terao J. Lipidomic Analysis for Lipid Peroxidation-Derived Aldehydes Using Gas Chromatography-Mass Spectrometry. Chem. Res. Toxicol. 2007, 20, 99–107. 10.1021/tx060199e. 17226932

[ref18] Schaur R. J. Basic Aspects of the Biochemical Reactivity of 4-Hydroxynonenal.. Mol. Aspects Med. 2003, 24, 149–159. 10.1016/S0098-2997(03)00009-8. 12892992

[ref19] Csala M.; Kardon T.; Legeza B.; Lizák B.; Mandl J.; Margittai É.; Puskás F. On the Role of 4-Hydroxynonenal in Health and Disease. Biochim. Biophys. Acta, Mol. Basis Dis. 2015, 1852 (5), 826–838. 10.1016/j.bbadis.2015.01.015. 25643868

[ref20] Eckl P. M. Genotoxicity of HNE. Mol. Aspects Med. 2003, 24, 161–165. 10.1016/S0098-2997(03)00010-4. 12892993

[ref21] Zhong H.; Yin H. Role of Lipid Peroxidation Derived 4-Hydroxynonenal (4-HNE) in Cancer: Focusing on Mitochondria. Redox Biol. 2015, 4, 193–199. 10.1016/j.redox.2014.12.011. 25598486 PMC4803793

[ref22] Lopachin R. M.; Gavin T. Molecular Mechanisms of Aldehyde Toxicity: A Chemical Perspective. Chem. Res. Toxicol. 2014, 27, 1081–1091. 10.1021/tx5001046. 24911545 PMC4106693

[ref23] Baba S. P.; Hoetker J. D.; Merchant M.; Klein J. B.; Cai J.; Barski O. A.; Conklin D. J.; Bhatnagar A. Role of Aldose Reductase in the Metabolism and Detoxification of Carnosine-Acrolein Conjugates. J. Biol. Chem. 2013, 288 (39), 28163–28179. 10.1074/jbc.M113.504753. 23928303 PMC3784727

[ref24] Yeum K.-J.; Orioli M.; Regazzoni L.; Carini M.; Rasmussen H.; Russell R. M.; Aldini G. Profiling Histidine Dipeptides in Plasma and Urine after Ingesting Beef, Chicken or Chicken Broth in Humans. Amino Acids 2010, 38, 847–858. 10.1007/s00726-009-0291-2. 19381778

[ref25] Ludolph A. ALS - A Multisystem Degeneration. J. Neurol. Sci. 2017, 381, 42 10.1016/j.jns.2017.08.173.

[ref26] Shefner J. M.; Musaro A.; Ngo S. T.; Lunetta C.; Steyn F. J.; Robitaille R.; De Carvalho M.; Rutkove S.; Ludolph A. C.; Dupuis L. Skeletal Muscle in Amyotrophic Lateral Sclerosis. Brain 2023, 146 (11), 4425–4436. 10.1093/brain/awad202. 37327376 PMC10629757

[ref27] Howland D. S.; Liu J.; She Y.; Goad B.; Maragakis N. J.; Kim B.; Erickson J.; Kulik J.; De Vito L.; Psaltis G.; Degennaro L. J.; Cleveland D. W.; Rothstein J. D Focal Loss of the Glutamate Transporter EAAT2 in a Transgenic Rat Model of SOD1Mutant-Mediated Amyotrophic Lateral Sclerosis (ALS). Proc. Natl. Acad. Sci. U. S. A. 2002, 99 (580), 1604–1609. 10.1073/pnas.032539299. 11818550 PMC122237

[ref28] Chaves-Filho A. B.; Pinto I. F. D.; Dantas L. S.; Xavier A. M.; Inague A.; Faria R. L.; Medeiros M. H. G.; Glezer I.; Yoshinaga M. Y.; Miyamoto S. Alterations in Lipid Metabolism of Spinal Cord Linked to Amyotrophic Lateral Sclerosis. Sci. Rep. 2019, 9, 11642 10.1038/s41598-019-48059-7. 31406145 PMC6691112

[ref29] Carvalho V. H.; Oliveira A. H. S.; de Oliveira L. F.; Silva R. P.; Di Mascio P.; Gualano B.; Artioli G. G.; Medeiros M. H. G Exercise and β -Alanine Supplementation on Carnosine-Acrolein Adduct in Skeletal Muscle. Redox Biol. 2018, 18, 222–228. 10.1016/j.redox.2018.07.009. 30053728 PMC6077140

[ref30] Falletti O.; Cadet J.; Favier A.; Douki T. Trapping of 4-Hydroxynonenal by Glutathione Efficiently Prevents Formation of DNA Adducts in Human Cells.. Free Radicals Biol. Med. 2007, 42, 1258–1269. 10.1016/j.freeradbiomed.2007.01.024. 17382206

[ref31] Blair I. A. Endogenous Glutathione Adducts. Curr. Drug Metab. 2006, 853–872. 10.2174/138920006779010601. 17168687

[ref32] Bispo V. S.; De Arruda Campos I. P.; Di Mascio P.; Medeiros M. H. G. Structural Elucidation of a Carnosine-Acrolein Adduct and Its Quantification in Human Urine Samples. Sci. Rep. 2016, 6, 1–5. 10.1038/srep19348. 26783107 PMC4726056

[ref33] Orioli M.; Aldini G.; Beretta G.; Facino R. M.; Carini M. LC-ESI-MS/MS Determination of 4-Hydroxy-Trans-2-Nonenal Michael Adducts with Cysteine and Histidine-Containing Peptides as Early Markers of Oxidative Stress in Excitable Tissues.. J. Chromatogr. B Anal. Technol. Biomed Life Sci. 2005, 827 (1), 109–118. 10.1016/j.jchromb.2005.04.025. 15916929

[ref34] Simpson E. P.; Henry Y. K.; Henkel J. S.; Smith R. G.; Appel S. H. Increased Lipid Peroxidation in Sera of ALS Patients A Potential Biomarker of Disease Burden. Neurology 2004, 62, 1758 10.1212/wnl.62.10.1758. 15159474

[ref35] Stuerenburg H. J.; Kunze K. Concentrations of Free Carnosine (a Putative Membrane-Protective Antioxidant) in Human Muscle Biopsies and Rat Muscles. Arch. Gerontol. Geriatr. 1999, 29 (2), 107–113. 10.1016/S0167-4943(99)00020-5. 15374064

[ref36] Boldyrev A. A.; Aldini G.; Derave W. Physiology and Pathophysiology of Carnosine. Physiol. Rev. 2013, 93, 1803–1845. 10.1152/physrev.00039.2012. 24137022

[ref37] Esterbauer H.; Zöllner H.; Scholz N. Reaction of Glutathione with Conjugated Carbonyls. Zeitschrift für Naturforsch. C 1975, 30, 466–473. 10.1515/znc-1975-7-808. 241172

[ref38] Zhao J.; Posa D. K.; Kumar V.; Hoetker D.; Kumar A.; Ganesan S.; Riggs D. W.; Bhatnagar A.; Wempe M. F; Baba S. P Carnosine Protects Cardiac Myocytes against Lipid Peroxidation Products. Amino Acids 2019, 51 (1), 123–138. 10.1007/s00726-018-2676-6. 30449006 PMC6377314

[ref39] Loeffler J.; Picchiarelli G.; Dupuis L.; Aguilar J. G. De The Role of Skeletal Muscle in Amyotrophic Lateral Sclerosis. Brain Pathol. 2016, 26, 227–236. 10.1111/bpa.12350. 26780251 PMC8029271

[ref40] Mastrogiovanni M.; Trostchansky A.; Naya H.; Dominguez R.; Marco C.; Povedano M.; López-Vales R.; Rubbo H. HPLC-MS/MS Oxylipin Analysis of Plasma from Amyotrophic Lateral Sclerosis Patients. Biomedicines 2022, 10, 674 10.3390/biomedicines10030674. 35327476 PMC8945419

[ref41] Spiteller P.; Kern W.; Reiner J.; Spiteller G. Aldehydic Lipid Peroxidation Products Derived from Linoleic Acid. Biochim. Biophys. Acta 2001, 1531, 188–208. 10.1016/S1388-1981(01)00100-7. 11325611

