# Supplementary material for: Quantitative Analysis of Glutathione and Carnosine Adducts with 4-Hydroxy-2-nonenal in Muscle in a hSOD1G93A ALS Rat Model
Source: Chem Res Toxicol. 2024 Jul 27;37(8):1306–14. doi: 10.1021/acs.chemrestox.4c00052 (PMC11337210; doi:10.1021/acs.chemrestox.4c00052)
Supplement: Supplementary file 1 — tx4c00052_si_001.pdf [file tx4c00052_si_001.pdf]

Quantitative analysis of glutathione and carnosine adducts with 4-hydroxy-2-nonenal in muscle in a hSOD1^{G93A} ALS rat model

*Pablo V. M. Reis†, Bianca S. Vargas†, Rafael A. Rebelo, Mariana P. Massafera, Fernanda M. Prado, Hector Oreliana, Henrique V. de Oliveira, Florêncio P. Freitas, Graziella E. Ronsein, Sayuri Miyamoto, Paolo Di Mascio, Marisa H. G. Medeiros**

Departamento de Bioquímica, Instituto de Química, Universidade de São Paulo, São Paulo, SP, Brazil

Table of contents:

Page 2: **Table S1.** Mass Spectrometry parameters for detection and quantification of glutathione analytes. Quantification transitions are highlighted.

Page 2: **Table S2.** Mass Spectrometry parameters for detection and quantification of carnosine analytes. Quantification transitions are highlighted.

Page 2: **Table S3.** Mass Spectrometry parameters for detection and quantification of free 4-hydroxynonenal analytes. Quantification transitions are highlighted.

Page 3: **Figure S1.** GS-NEM and GSSG ratio in the analyzed tissues.

Page 4: Free 4-hydroxynonenal extraction from rat muscle tissue.

Page 4: Quantification of HNE-DNPH in rat tissues.

Page 6: **Figure S2**. Analysis of HNE-DNPH and HNEd_{11}-DNPH (internal standard) derivatives in muscle tissue. HPLC/ESI$^-$/MS-MS analyses carried out in the negative mode.

Table S1. Mass Spectrometry parameters for detection and quantification of glutathione analytes. Quantification transitions are highlighted.

Analyte	Percursor Ion (*m/z*)	Fragment (*m/z*)	Declustering Potential (V)	Collision Energy (V)	Collision Cell Exit Potential (V)	Dwell Time (ms)	Entrance Potential (V)
GS-HNE	464.3	**446.1**	61	19	6	100	10
		308.0	61	13	12	100	10
[$^{13}C_2$,^{15}N-Gly]-GS-HNE	467.2	**449.0**	61	19	6	100	10
		311.0	61	13	12	100	10
GSSG	307.1	**355.3**	51	21	8	100	10
		130.0	51	17	10	100	10
[($^{13}C_2$,^{15}N-Gly)$_2$]-GSSG	310.1	**360.9**	51	21	8	100	10
		130.0	51	17	10	100	10
GS-NEM	433.0	**304.3**	71	21	14	100	10
		201.3	71	31	10	100	10
		158.0	71	55	8	100	10

Table S2. Mass Spectrometry parameters for detection and quantification of carnosine analytes. Quantification transitions are highlighted.

Analyte	Percursor Ion (*m/z*)	Fragment (*m/z*)	Declustering Potential (V)	Collision Energy (V)	Collision Cell Exit Potential (V)	Dwell Time (ms)	Entrance Potential (V)
Car	227.0	**110.0**	36	31	12	200	10
		156.0	36	21	10	200	10
Card_4	231.0	**110.0**	36	31	12	200	10
		156.0	36	21	10	200	10
Car-HNE	383.2	**366.2**	96	25	26	60	10
		312.2	96	29	22	60	10
		266.0	96	37	20	60	10
Car-HNEd_{11}	394.3	**377.2**	96	25	26	60	10
		323.3	96	29	22	60	10
		277.0	96	37	20	60	10

Table S3. Mass Spectrometry parameters for detection and quantification of free 4-hydroxynonenal analytes. Quantification transitions are highlighted.

Analyte	Percursor Ion (*m/z*)	Fragment (*m/z*)	Declustering Potential (V)	Collision Energy (V)	Collision Cell Exit Potential (V)	Dwell Time (ms)	Entrance Potential (V)
HNE-DNPO	335.2	**167.0**	-50	-22	-13	200	-10
		162.8	-50	-26	-17	200	-10
HNEd_{11}-DNPO	346.2	**167.0**	-50	-22	-13	200	-10
		162.8	-50	-26	-17	200	-10

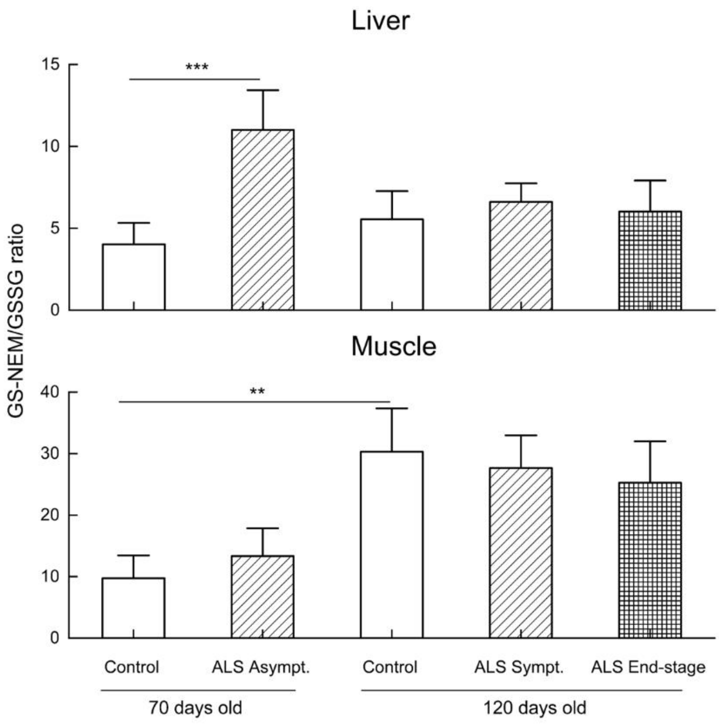

Figure S1. GS-NEM and GSSG ratio in the analyzed tissues.

Free 4-hydroxynonenal extraction from rat muscle tissue

The muscle tissue (20 mg) was homogenized in 150 µL of sodium acetate buffer 100 mM, pH 5.0 containing EDTA 5 mM. To the homogenized mixture it was added 100 µL of 10% sodium dodecyl sulfate and 150 µL of 0.2% butyl-hydroxytoluene in ethanol. The samples were incubated in ice for an hour and centrifuged at 3.500 RPM for 10 min. 100 µL were collected for protein quantification using Pierce™ BCA Protein Assay Kit, Thermo Scientific™. To the supernatant 700 µL of ethanol were added. The samples were then centrifuged at 14.000 RPM, 4 °C for 10 min. To 500 µL of the supernatant 100 µL of 2,4-dinitrophenylhydrazine (DNPH) 20 mM in HCl 1.0 M (ethanolic solution).

The samples were kept for 2 h at 37 °C, under agitation at 500 RPM. After this time the analyte was extracted with 1:1 chloroform (%v/v), the organic phase was collected and dried under N_2 and dissolved in 200 µL acetonitrile. For the analysis in the HPLC-MS/MS system, the samples were 10 times diluted, HNEd_{11} was added as an internal standard and 20 µL was injected for the quantification of the aldehyde-DNPH.

Quantification of HNE-DNPH in rat tissues.

Online HPLC/ESI$^-$/MS-MS analyses were carried out in the negative mode and detection was conducted on a triple quadrupole mass spectrometer API 6500 (Sciex, Washington D.C, WA), using selected reaction monitoring (SRM). The turbo ionspray voltage was kept at -4500 V, curtain gas at 20 psi and nebulizer and auxiliary gas at 50 psi. The temperature was set at 500°C, and nitrogen pressure in the collision cell was adjusted to high. An Agilent HPLC system (Agilent Technologies, Santa Clara, CA) equipped with an autosampler (1200 High performance) cooled to 4ºC, a column oven

set at 37ºC (1200 G1216B) with automated high pressure flow switching valve, a 1200 Binary Pump SL (1200 G1310A) and a Shimadzu 10-AVp Isocratic Pump (Shimadzu, Tokyo, Japan) were used for sample injection using a Luna C18 column (150 mm x 2.0 mm i.d., 3 μm particle size; Phenomenex, Torrance, CA, USA), the flow rate was 300 μL/min. Mobile phases were 0.1% (v/v) ammonium acetate (A), acetonitrile (B) and a mixture of A:B 1:1 (v/v) (C). Prior to use, solutions were filtered through a 0.1 μm PVDF membrane (Millipore, Bedford, MA). The adducts were eluted from the column according to the following method: from 0 to 10 min, 20% B; from 10 to 11 min, 20 to 98% B; from 11 to 17 min, 98% B; from 17 to 18 min; 98 to 20% B; from 18 to 25 min, 20% B to re-equilibrate the column. A high-pressure flow switching valve composed of 2-positions and 6-ports was inserted after the column. The valve discarded the eluent from the column until 3 min of run while kept the mass spectrometer supplied with solvent C at a constant flow of 100 μL/min using a Shimadzu 10-AVp Isocratic Pump. After 3 min of run, the valve switched position allowing the eluate from the column to enter the mass spectrometer. After 10 min of run, the valve switched back to waste position. A calibration curve for quantification was created using the reaction of acetaldehyde and a solution 1:1 (% v/v) of 20 mM 2,4-dinitrophenylhydrazine in 10 M HCl, HNEd_{11} was used as an internal standard.

The calculated level of HNE in muscle tissue was 0.3 ± 0.01 nmol/mg protein,

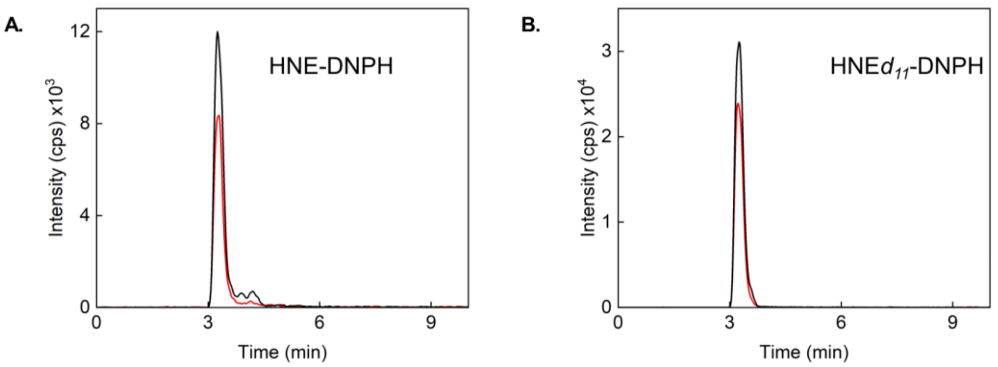

Figure S2. Analysis of HNE-DNPH and HNEd_{11}-DNPH (final concentration, 50 fmol/ µL) (internal standard) derivatives in muscle tissue. HPLC/ESI$^-$/MS-MS analyses
